# Reliability-Aware Image–Wireless Fusion for Through-Wood Termite Detection

**DOI:** 10.3390/s26154859

**Published:** 2026-08-01

**Authors:** Wei Zhang, Xiangshu Qi, Qinglong Tian, Ziqian Ling, Yi Cao, Youxi Zhang, Alex Qi

**Affiliations:** 1 College of Computer Science and Engineering, Changsha University, Changsha 410022, China; 2 Graduate School of Computer and Information Sciences, Hosei University, Tokyo 102-8160, Japan; 3 Pontosense Inc., Kitchener, ON N2H 5G5, Canada

**Keywords:** termite detection, image–wireless fusion, mmWave radar, Wi-Fi CSI, reliability-aware fusion, heritage timber inspection

## Abstract

Termite infestation poses a critical threat to ancient timber structures because hidden in-wood activity can cause progressive structural decay before visible surface symptoms appear. Through-wood termite detection remains challenging because termite-induced electromagnetic responses are weak, small-scale, and vulnerable to timber attenuation and multipath propagation. To address this problem, this study presents one of the first investigations to formulate through-wood termite detection as a multi-frequency wireless sensing and image–wireless fusion problem for non-destructive heritage timber inspection. We propose a Reliability-Aware Image–Wireless Fusion Network (RA-IWFNet), in which the image branch captures high-resolution surface-level visual cues while the dual-band wireless branch integrates complementary mmWave radar micro-motion responses and Wi-Fi Channel State Information (CSI) channel variations. A learnable temperature-scaled fusion gate estimates input-dependent image and wireless contributions and constructs a normalized fused representation for four-class recognition, including Termite, Lyctidae, Human, and None. Here, reliability is operationally defined as learned input-adaptive relative modality contribution rather than explicit uncertainty or signal-quality estimation. RA-IWFNet is evaluated under two complementary protocols: a field-motivated protocol with joint visual and wireless degradation and a synchronized verification protocol using physically co-acquired multimodal samples. Across repeated training runs, RA-IWFNet achieves 81.91±1.33% accuracy and 81.96±1.27% Macro-F1 under field-mixed visual degradation and moderate wireless degradation. On the synchronized verification subset, gated fusion achieves 91.53±2.44% accuracy and 91.62±2.44% Macro-F1, yielding higher mean performance than single-modality and non-adaptive fusion baselines. Feature-space, error-correction, and gate-temperature analyses further support the effectiveness of adaptive modality integration. These results provide controlled laboratory feasibility evidence and suggest that multi-frequency wireless sensing combined with adaptive image–wireless fusion offers a promising non-invasive pathway toward practical through-wood termite inspection in heritage timber structures.

## 1. Introduction

Termite infestation is a persistent and geographically expanding threat to heritage timber structures because hidden in-wood biological activity can cause progressive internal decay before visible surface symptoms appear [1,2]. Although termite baiting and localized colony management strategies, including chitin synthesis inhibitor baits, have improved colony-level control [3,4,5], heritage timber preservation still requires non-destructive sensing methods capable of identifying hidden biological activity before severe structural damage occurs.

Camera-sensor imaging and contactless wireless sensing provide two complementary pathways for non-invasive termite inspection. Camera-sensor inspection captures high-resolution surface-level visual information, including insect morphology, wood texture changes, mud tubes, frass, and surface damage. These visual cues are useful for fine-grained category discrimination, such as distinguishing termites from morphologically similar wood-boring insects. Recent deep-learning-based visual detection studies have also shown the potential of object detection models for termite-related damage recognition [6]. However, optical sensing is limited to surface-visible evidence and cannot directly observe early-stage infestation hidden inside timber.

Wireless sensing can provide through-wood perception beyond surface-visible observations. Within this modality, different frequency bands capture complementary physical responses. Millimeter-wave (mmWave) radar uses short wavelengths and is sensitive to fine-grained insect-induced micro-motion responses, but its signals can be weakened by timber attenuation, scattering, and multipath propagation [7]. Wi-Fi Channel State Information (CSI), by contrast, provides penetrative channel-level variations at longer wavelengths, but its representation of termite-related activity is relatively coarse and less sensitive to fine-scale biological motion signatures [8]. Therefore, neither camera-only nor wireless-only sensing is sufficient as a standalone solution for heritage timber inspection under non-ideal conditions. This limitation highlights the need for an integrated framework that combines the high-resolution surface discrimination of camera-sensor images with the hidden-activity perception of multi-frequency wireless sensing.

To develop a practical image–wireless termite inspection system, three key challenges must be addressed.

1. **Through-wood perception of weak biological targets.** Existing electromagnetic-wave-based sensing studies are mainly designed for human-centric or object-scale perception tasks. Adapting such methods to through-wood termite detection is difficult because termite-induced responses are weak, localized, and easily affected by timber attenuation and environmental interference. mmWave radar is sensitive to fine-grained biological micro-motion, but its response may become unstable under dense wood, sensor-target distance changes, and multipath propagation. Wi-Fi CSI provides more penetrative channel-level responses, but its features are relatively coarse and may be insufficient for isolating small-scale biological signatures.
2. **Taxonomic ambiguity and modality-specific sensing limitations.** Distinguishing active termites from visually or biologically similar wood-boring insects, such as hlLyctidae, remains challenging. Wireless signals can capture hidden activity but lack the high-resolution morphological details needed for fine-grained pest category discrimination or for separating target activity from non-target interference such as human movement. Conversely, camera-sensor images provide rich surface morphology but cannot directly observe internal activity. In addition, on-site inspection images may suffer from illumination variation, shadow, blur, compression, partial occlusion, and cropping, causing image-only models to lose critical semantic details or produce false-positive termite predictions.
3. **Input-adaptive integration of heterogeneous cues.** Image and wireless modalities rely on different physical sensing mechanisms and exhibit different degradation patterns. Under field-motivated non-ideal inspection conditions, the useful contribution of each modality is input-dependent. Visual cues can be degraded by poor image acquisition or hidden infestation, while wireless signals can fluctuate with timber thickness, material heterogeneity, moisture variation, sensor placement, and multipath propagation. Simple feature concatenation or static modality weighting may propagate errors when a degraded modality dominates the fused representation.

Our study is motivated by the physical sensing insight that through-wood termite detection cannot be adequately addressed as either a conventional visual pest-recognition task or a generic RF sensing task. In preliminary sensing experiments, mmWave radar showed sensitivity to fine-grained biological micro-motion, but its response became less stable when the propagation path was affected by timber attenuation, sensor-target distance, and room-dependent multipath. Wi-Fi CSI provided complementary penetrative channel-level responses, but its representation alone was insufficient for consistently separating termite-related activity from visually or biologically similar interference. At the same time, camera-sensor images provided high-resolution surface morphology and contextual cues, which are valuable for distinguishing termites from similar insects, human interference, and no-target background conditions. However, camera-sensor images cannot directly observe hidden in-wood activity. These observations led us to formulate through-wood termite detection as a **multi-frequency wireless sensing and image–wireless fusion problem**, rather than as a single-modality pest-recognition task. To the best of our knowledge, this work is among the first attempts to apply the fusion of mmWave radar, Wi-Fi CSI, and camera-sensor images to practical termite-related recognition inside timber structures.

**Figure 1:**
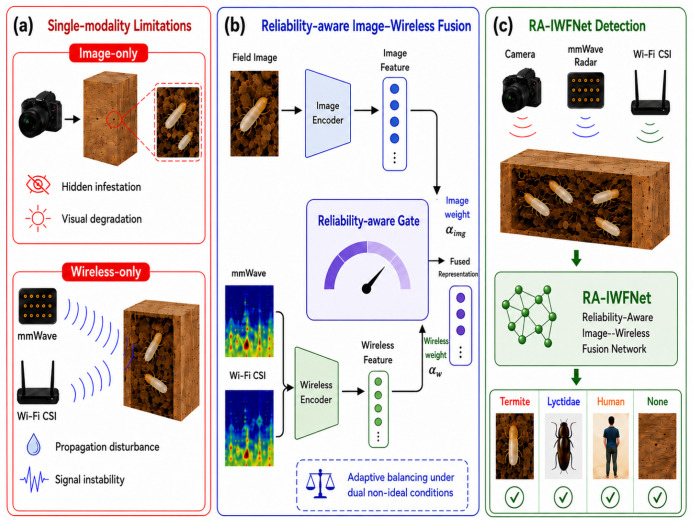
Reliability-aware image–wireless fusion for through-wood termite detection. (**a**) Single-modality sensing limitations: image-only sensing can capture high-resolution surface visual cues but fails under hidden infestation and visual degradation, while wireless-only sensing provides through-wood perception but is affected by attenuation, noise, and multipath-related feature variation. (**b**) Reliability-aware image–wireless fusion: field-motivated visual observations and dual-band wireless signals are encoded into image and wireless features, which are adaptively balanced by a reliability-oriented fusion gate to form a fused representation. (**c**) RA-IWFNet detection: the fused representation supports four-class termite-related recognition, including Termite, Lyctidae, Human, and None, under visual degradation and wireless signal disturbance.

**Figure 2:**
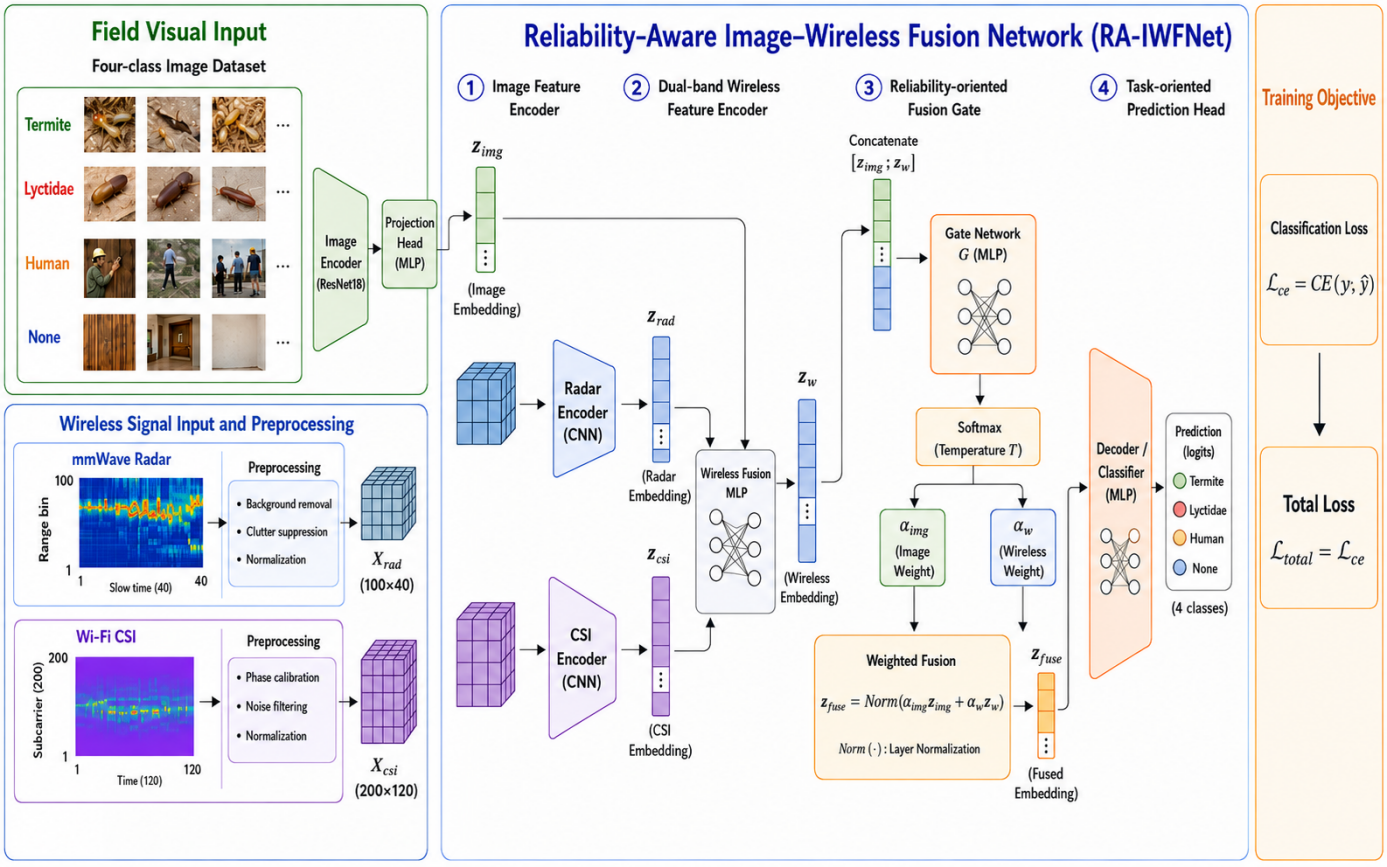
Architecture of the proposed Reliability-Aware Image–Wireless Fusion Network (RA-IWFNet). The model extracts image features from field-motivated visual observations and wireless features from mmWave radar and Wi-Fi CSI. A reliability-oriented gate estimates image and wireless weights and produces a fused representation for four-class termite-related recognition. The arrows indicate the data and feature-processing flow, while the colors distinguish the image, radar, CSI, fusion, and prediction modules.

To address these challenges, we propose a **Reliability-Aware Image–Wireless Fusion Network** (**RA-IWFNet**) for non-destructive termite detection in heritage timber inspection, as illustrated in Figure 1b and Figure 2. The proposed framework integrates camera-sensor images, mmWave radar, and Wi-Fi CSI into a unified multimodal sensing model. The image branch extracts high-resolution surface-level visual embeddings, while the wireless branch jointly encodes radar-based micro-motion responses and CSI-based through-wood channel variations. A reliability-oriented fusion gate then estimates the relative contribution of image and wireless features and generates an adaptive fused representation for four-class prediction.

Different from fixed-weight fusion or late decision fusion, RA-IWFNet is designed to perform feature-level image–wireless fusion with input-adaptive modality weighting. When visual observations are degraded or hidden in-wood activity cannot be directly observed from the surface, wireless features provide through-wood sensing evidence. When wireless signals are disturbed or fine-grained category discrimination is required, image features provide high-resolution appearance cues. This adaptive mechanism reduces over-reliance on either modality and improves robustness when visual observations and wireless signals are simultaneously degraded.

The main contributions of this article are summarized as follows.

1. **An early exploration of multi-frequency wireless and image fusion for weak through-wood biological sensing.** To the best of our knowledge, this work presents one of the first image–wireless termite-inspection frameworks that jointly uses mmWave radar, Wi-Fi CSI, and camera-sensor images for non-destructive recognition inside timber structures. Different from existing wireless sensing studies that mainly focus on human-centric or object-scale perception, this work addresses small biological activity hidden inside wood, where the sensing response is weak, localized, and vulnerable to timber attenuation and multipath propagation. The results show that radar-based micro-motion cues, CSI-based penetrative channel variations, and camera-sensor surface morphology provide complementary evidence for termite-related recognition.
2. **An image–wireless inspection framework motivated by complementary sensing limitations.** We introduce camera-sensor images as a complementary modality to dual-band wireless sensing. The image branch provides high-resolution surface morphology and contextual cues for distinguishing termites from visually similar insects, human interference, and no-target background conditions, while the wireless branch provides through-wood activity information from hidden biological responses. This design addresses the practical need to recognize Termite, Lyctidae, Human, and None under visual degradation and wireless propagation disturbance.
3. **A reliability-aware image–wireless fusion mechanism for input-adaptive modality weighting.** We develop RA-IWFNet, which learns adaptive image and wireless modality weights through a reliability-oriented gate. The model constructs a fused representation by balancing surface-level visual cues and through-wood wireless cues for each input instance, reducing over-reliance on either modality under visual degradation and wireless signal disturbance.
4. **Comprehensive evaluation under field-motivated and synchronized image–wireless settings.** We evaluate the proposed framework under image-only visual degradation, wireless-only signal degradation, joint image–wireless degradation, and synchronized image–wireless verification settings. The results include baseline comparison, component ablation, prediction-level error correction, three-run evaluation, feature-space separability analysis, gate-temperature sensitivity, and normalized confusion matrix evaluation.

The remainder of this article is organized as follows. Section 2 reviews related work. Section 3 presents the theoretical foundations and signal modeling. Section 4 introduces the proposed RA-IWFNet methodology. Section 5 describes the experiments and analyses. Section 6 concludes this article.

## 2. Related Work

### 2.1. Termite Monitoring and Non-Destructive Inspection

Termite detection and management have long been important topics in structural pest control. Subterranean termite infestations remain difficult to identify because colonies often forage within soil, wood, or concealed structural cavities before visible damage appears. Recent reviews of termite baiting show that colony-level management has become a major component of modern termite control, especially because chitin synthesis inhibitor baits can suppress or eliminate colonies while reducing chemical input into the environment [3,4]. Field studies on *Coptotermes gestroi* further show that above-ground bait stations can be effective when active infestation sites are located, while in-ground interception may fail in some environments [5]. These studies highlight the practical importance of routine inspection and early activity discovery.

At the same time, invasive termite studies indicate that termite-related structural risks are expanding geographically. Molecular identification of *Reticulitermes grassei* outside its known distribution emphasizes the need for early warning and monitoring programs in susceptible regions [2]. More broadly, invasive termite species are becoming major urban pests because human-mediated transportation, urban adaptation, and delayed discovery can allow colonies to remain undetected for long periods [1]. These findings motivate the development of non-destructive sensing methods that can detect hidden termite activity in timber before severe structural damage occurs.

### 2.2. Image-Based Pest and Termite Detection

Image-based pest recognition has been widely studied in agricultural, ecological, and infrastructure monitoring. Deep convolutional networks and vision transformers have been used to identify insects, pests, and damage patterns from RGB images. For termite-related applications, recent work proposed MCD-YOLOv8 for detecting soil-dwelling termite damage in hydraulic engineering environments, showing that improved object detection models can enhance the intelligent identification of termite activity traces [6]. These studies demonstrate the usefulness of camera-sensor images for visible pest monitoring.

For timber inspection, camera-sensor images provide high-resolution surface-level information, including insect morphology, body texture, mud tubes, frass, surface damage, and wood texture changes. These fine-grained visual cues are valuable for distinguishing termites from visually similar insects and other surface-level interference. However, image-based methods are fundamentally limited when biological activity is hidden inside timber. In ancient timber inspection, termites may remain concealed inside wooden members, and surface images may contain only weak, indirect, or incomplete evidence. Moreover, field images may be degraded by lighting disturbance, blur, compression, occlusion, and cropping. Therefore, image-only recognition can be effective when targets or damage traces are visible, but it is insufficient for reliable through-wood detection of hidden termite activity.

### 2.3. Wireless Sensing for Contactless Perception

Electromagnetic-wave-based sensing has been widely explored for contactless perception, including mmWave radar, Wi-Fi CSI, RFID, FMCW radar, and emerging intelligent-surface-assisted sensing. For human-centric sensing, mmWave radar has been used for facial expression recognition [9], blood pressure monitoring [10], gesture recognition [11], stress monitoring [12], and sleep posture recognition [13]. Wi-Fi CSI has also been adopted for respiration monitoring [14], gesture tracking [15], and object size measurement [16] due to its sensitivity to channel variations and low-cost deployment. Recent studies further extend wireless sensing to hand rehabilitation tracking using CSI [17], synthetic RF data generation for human activity recognition using RFID, CSI, and FMCW radar [18], federated domain adaptation for mmWave-based human activity recognition [19], and intelligent-surface-assisted multi-target detection [20].

Although these works demonstrate the capability of EM-wave sensing, their targets are primarily human bodies, limbs, gestures, vital signs, or generic spatial objects. Directly applying such methods to termite detection is challenging because termite-related responses are weaker, smaller, and often hidden inside timber. mmWave radar can capture fine-grained micro-motion responses, whereas Wi-Fi CSI can provide penetrative channel-level variations under wood occlusion. However, wireless sensing alone has limited access to high-resolution surface morphology, which is important for distinguishing termites from visually similar insects or surface-level interference. A few pioneering studies have investigated wireless termite sensing, including radar-based termite detection and microwave-based identification of termites in dry wood [7,21]. Nevertheless, robust termite detection that jointly exploits surface-level visual discrimination and hidden through-wood wireless sensing remains insufficiently studied.

### 2.4. Multimodal Fusion and Research Gap

Multimodal learning provides a useful framework for integrating heterogeneous sensing cues. Feature-level multimodal fusion methods, such as tensor-based fusion, introduced the idea of learning joint representations from heterogeneous modality embeddings [22]. General multimodal learning surveys further show that multimodal systems involve representation learning, alignment, fusion, translation, and co-learning, rather than simple early or late fusion [23]. More recent studies have extended this direction toward missing-modality robustness, quality-aware fusion, dynamic modality weighting, and adverse-condition sensor fusion [24,25,26,27].

These studies provide important methodological foundations for adaptive multimodal fusion. Table 1 summarizes the positioning of RA-IWFNet relative to representative multimodal fusion studies. However, most of them are developed and validated in domains such as affect recognition, multimedia understanding, anomaly detection, autonomous driving, or general sensor perception. The sensing assumptions in termite inspection are different. Camera-sensor images provide high-resolution surface morphology and contextual information, mmWave radar captures fine-grained micro-motion responses, and Wi-Fi CSI captures penetrative channel-level variations. The challenge is therefore not only to fuse heterogeneous features but also to combine surface-level visual discrimination with hidden-activity wireless perception for small biological activity inside timber.

Compared with existing multimodal fusion studies, the contribution of RA-IWFNet is not a claim of a completely new generic gating equation. Instead, this work provides a task-instantiated image–wireless sensing framework for a previously underexplored inspection problem. To the best of our knowledge, few studies have integrated camera-sensor images, mmWave radar, and Wi-Fi CSI for through-wood termite detection. The novelty of this work therefore lies in formulating termite inspection as a multi-frequency wireless and image-fusion problem, and in validating an input-adaptive fusion strategy that balances surface-level visual cues and hidden through-wood wireless cues under visual degradation and wireless signal disturbance. This formulation addresses a gap that is not covered by image-only pest recognition, human-centric wireless sensing, or generic multimodal fusion studies alone.

## 3. Theoretical Foundations and Signal Modeling

This section establishes the sensing rationale of the proposed image–wireless termite detection framework. Termite-related activity inside timber can produce both coarse-grained channel fluctuations and fine-grained micro-motion responses, while surface observations can provide high-resolution morphological evidence when insects or traces are visible. Wi-Fi CSI, mmWave radar, and camera-sensor images therefore provide complementary sensing cues: CSI captures penetrative channel-level variations, mmWave radar captures fine-scale motion responses, and camera-sensor images provide high-resolution surface morphology and semantic context. These complementary but condition-dependent cues motivate the reliability-aware image–wireless fusion design of RA-IWFNet.

### 3.1. Coarse-Grained Through-Wood Sensing: Wi-Fi CSI

Wi-Fi CSI provides a long-wavelength wireless sensing modality with relatively stronger penetration capability than mmWave radar in wooden-obstacle sensing scenarios. In a timber inspection environment, the received channel response at subcarrier frequency *f* and time *t* can be modeled as the superposition of quasi-static structural paths and dynamic biological paths:

(1) $$\begin{array}{cc} H(f,t)\; & =\sum_{p\in L_{s}}\alpha_{p}e^{-j2\pi f\tau_{p}}+\sum_{q\in L_{d}}\alpha_{q}\left(t\right)e^{-j2\pi f\tau_{q}\left(t\right)}+N\left(f,t\right), \end{array}$$

where H(f,t) denotes the complex CSI measurement, $L_{s}$ and $L_{d}$ denote the sets of quasi-static and dynamic paths, respectively, $\alpha_{p}$ and $\tau_{p}$ are the attenuation coefficient and propagation delay of the *p*-th static path, $\alpha_{q}\left(t\right)$ and $\tau_{q}\left(t\right)$ are the time-varying attenuation coefficient and delay of the *q*-th dynamic path, and N(f,t) represents noise and residual environmental interference.

Biological activity inside timber can introduce temporal fluctuations into the channel response. These fluctuations can be illustrated by the temporal variance of CSI magnitude within a sliding window:

(2) $$\sigma_{\left|H\right|}^{2}\left(t\right)=\frac{1}{W}\sum_{k=t-W+1}^{t}{\left(\left|H\left(f,k\right)\right|-{\bar{\left|H(f)\right|}}_{t}\right)}^{2},$$

where *W* is the temporal window length, |H(f,k)| is the CSI magnitude at time index *k*, and ${\bar{\left|H(f)\right|}}_{t}$ denotes the mean CSI magnitude within the current window. A larger $\sigma_{\left|H\right|}^{2}\left(t\right)$ indicates stronger time-varying channel disturbance.

In this work, Equations (1) and (2) are used to explain why the CSI branch should preserve temporal channel variations, rather than to define a handcrafted physical-parameter feature. Specifically, termite-related activity may introduce weak time-varying disturbances into the complex CSI response. Therefore, the CSI stream in RA-IWFNet keeps the complex channel information and constructs a temporal channel-variation map from the real and imaginary components of $H_{i}\left(f_{m},t\right)$, as operationalized in Section 4.3. The model does not explicitly estimate $\sigma_{\left|H\right|}^{2}\left(t\right)$ as a handcrafted feature. Instead, the learnable CSI encoder extracts discriminative channel-variation patterns from the preprocessed CSI feature map.

### 3.2. Fine-Grained Motion Sensing: mmWave Radar

mmWave radar complements CSI by providing short-wavelength phase sensitivity to fine-grained motion. For a small radial displacement Δd caused by biological motion, the corresponding phase change and micro-Doppler-sensitive frequency shift can be expressed as

(3) $$\Delta \phi =\frac{4\pi \Delta d}{\lambda },\;f_{D}\left(t\right)=\frac{1}{2\pi }\frac{d\phi (t)}{dt}=\frac{2}{\lambda }v_{\mathrm{bio}}\left(t\right),$$

where Δϕ is the received phase variation, λ is the radar wavelength, ϕ(t) is the time-varying radar phase, and $v_{\mathrm{bio}}\left(t\right)$ denotes the instantaneous radial velocity of the biological target.

Equation (3) indicates that mmWave radar is sensitive to weak insect-induced motion. In the preprocessing chain, the raw radar response is transformed along the fast-time dimension to obtain a range-dependent temporal response:

(4) $$R_{i}\left(r,t\right)=\left|\sum_{n=0}^{N-1}s_{i}\left(n,t\right)\exp\left(-j2\pi rn/N\right)\right|,$$

where $s_{i}\left(n,t\right)$ denotes the raw radar sample of the *i*-th measurement, *n* is the fast-time sampling index, *t* is the slow-time index, and *r* denotes the range-bin index.

In this work, Equation (4) motivates the construction of a range–temporal radar feature map in the model. The phase-change and micro-Doppler relations explain why this range–temporal representation can preserve fine-grained motion-sensitive responses. However, RA-IWFNet does not explicitly estimate micro-Doppler parameters as handcrafted features. Instead, the radar encoder learns discriminative motion-sensitive patterns from the preprocessed radar feature map, as defined in Section 4.3.

### 3.3. Camera-Sensor Image Sensing and Visual Reliability

Camera-sensor images provide high-resolution surface-level cues that are difficult to capture from wireless signals alone, including insect morphology, body texture, surface damage patterns, mud tubes, frass, wood texture changes, and contextual evidence for human or no-target conditions. These fine-grained visual cues are particularly useful for distinguishing termites from visually similar insects and surface-level interference.

Let $I_{i}$ denote the camera-sensor image of the *i*-th inspection sample. The image branch of RA-IWFNet later maps $I_{i}$ into the normalized image embedding $z_{i}^{\mathrm{img}}$. However, visual reliability is condition-dependent. In heritage inspection, field images may suffer from lighting disturbance, blur, compression, occlusion, cropping, and incomplete exposure. More importantly, camera-sensor images cannot directly observe biological activity hidden inside wooden members. Therefore, image features provide discriminative surface-level evidence but should not be treated as universally reliable. They need to be integrated with through-wood wireless cues in a reliability-aware manner.

### 3.4. Reliability-Aware Image–Wireless Fusion Rationale

The three sensing sources have complementary strengths and different sensing limitations. CSI provides penetrative but relatively coarse-grained channel variation, mmWave radar provides fine-grained micro-motion evidence, and camera-sensor images provide high-resolution surface morphology and semantic context. At the same time, each modality may become unreliable under non-ideal inspection conditions: images can be degraded by poor visual acquisition or may fail to expose hidden in-wood activity, while wireless signals can be degraded by attenuation, moisture variation, dielectric loss, sensor placement uncertainty, and multipath propagation.

Based on this rationale, RA-IWFNet encodes the radar feature map $X_{\mathrm{rad},i}$ and CSI feature map $X_{\mathrm{csi},i}$ into a unified wireless embedding $z_{i}^{w}$, and then constructs the final fused representation $z_{i}^{\mathrm{fuse}}$ by adaptively balancing the image embedding $z_{i}^{\mathrm{img}}$ and the wireless embedding $z_{i}^{w}$. This formulation provides the theoretical basis for the proposed model. When visual observations are degraded, the wireless representation can provide through-wood biological evidence. When wireless signals are disturbed or when fine-grained category discrimination is needed, the image representation can provide complementary surface-level appearance cues. Therefore, adaptive image–wireless fusion can produce a more robust representation than either image-only or wireless-only sensing in the tested inspection scenarios.

The signal models above are not used to explicitly estimate physical parameters in RA-IWFNet. Instead, they define the physical quantities that are operationalized in the model: $H_{i}\left(f_{m},t\right)$ is converted into a temporal CSI channel-variation map, the raw radar response $s_{i}\left(n,t\right)$ is transformed into the range-dependent temporal response $R_{i}\left(r,t\right)$ and then arranged as a range–temporal radar feature map, and $I_{i}$ is converted into an RGB visual tensor. These model inputs are then encoded, projected into a shared embedding space, and fused through the reliability-oriented gate.

## 4. Methodology

### 4.1. System Overview

The proposed system targets four-class termite detection in ancient timber structures, including Termite, Lyctidae, Human, and None. To improve detection robustness under both visual degradation and wireless propagation disturbance, we propose a **Reliability-Aware Image–Wireless Fusion Network** (**RA-IWFNet**). The overall architecture is shown in Figure 2.

Following the sensing formulation in Section 3, the *i*-th multimodal inspection sample is represented by three physically different observations, denoted as $O_{i}=\left\{I_{i},\;H_{i}\left(f_{m},t\right),\;s_{i}\left(n,t\right)\right\}$, where $I_{i}$ denotes the camera-sensor image, $H_{i}\left(f_{m},t\right)$ denotes the complex Wi-Fi CSI response defined by the channel model in Equation (1), and $s_{i}\left(n,t\right)$ denotes the raw mmWave radar response used to construct the range-dependent temporal response in Equation (4). For compact notation, $H_{i}$ denotes the full CSI measurement sequence ${\left\{H_{i}\left(f_{m},t\right)\right\}}_{m,t}$, and $s_{i}$ denotes the full radar measurement sequence ${\left\{s_{i}\left(n,t\right)\right\}}_{n,t}$.

RA-IWFNet converts these raw sensing observations into learnable input tensors through modality-specific preprocessing operators. Specifically, the image tensor, radar feature map, and CSI feature map are denoted as $X_{\mathrm{img},i}=P_{\mathrm{img}}\left(I_{i}\right)$, $X_{\mathrm{rad},i}=P_{\mathrm{rad}}\left(s_{i}\right)$, and $X_{\mathrm{csi},i}=P_{\mathrm{csi}}\left(H_{i}\right)$, respectively. Here, $P_{\mathrm{img}}\left(\cdot \right)$ denotes image resizing and RGB tensor conversion, $P_{\mathrm{rad}}\left(\cdot \right)$ denotes radar range–temporal feature-map construction, and $P_{\mathrm{csi}}\left(\cdot \right)$ denotes CSI real–imaginary separation and temporal reshaping.

The image branch extracts high-resolution surface-level appearance features from $X_{\mathrm{img},i}$, while the wireless branch extracts through-wood sensing features from $X_{\mathrm{rad},i}$ and $X_{\mathrm{csi},i}$. A reliability-oriented fusion gate then adaptively estimates the relative contribution of image and wireless modalities and generates a fused representation for final classification. The model supports three operating modes for experimental comparison: image-only recognition, wireless-only recognition, and image–wireless fusion. The proposed RA-IWFNet refers to the fusion mode, where visual and wireless features are jointly encoded and adaptively integrated by the reliability-oriented gate.

### 4.2. Image Feature Encoder

Given a camera-sensor image $I_{i}$, the image preprocessing operator converts it into an RGB tensor $X_{\mathrm{img},i}=P_{\mathrm{img}}\left(I_{i}\right)\in R^{3\times 224\times 224}$. The image encoder $E_{\mathrm{img}}\left(\cdot \right)$ extracts a high-level visual representation $h_{i}^{\mathrm{img}}=E_{\mathrm{img}}\left(X_{\mathrm{img},i}\right)$.

In our implementation, $E_{\mathrm{img}}\left(\cdot \right)$ is implemented using a ResNet18 backbone initialized without ImageNet pretraining. The extracted feature is projected into the shared embedding space as $z_{i}^{\mathrm{img}}=\mathrm{Norm}\left(\phi_{\mathrm{img}}\left(h_{i}^{\mathrm{img}}\right)\right)$, where $\phi_{\mathrm{img}}\left(\cdot \right)$ denotes the image projection layer, Norm(·) denotes $\ell_{2}$ normalization, and $z_{i}^{\mathrm{img}}\in R^{d}$ is the normalized image embedding.

The image branch provides high-resolution surface-level visual evidence, including insect morphology, body texture, visible surface traces, wood texture changes, and background appearance cues. These cues are useful for distinguishing termites from visually similar insects, human interference, and no-target background conditions. However, field images may be affected by lighting disturbance, blur, compression, partial occlusion, and incomplete visual exposure, and they cannot directly observe hidden activity inside wooden members. Therefore, image features are not treated as universally reliable and are further integrated with through-wood wireless features.

### 4.3. Dual-Band Wireless Feature Encoder

The dual-band wireless encoder operationalizes the CSI and radar sensing formulations in Section 3. For the CSI stream, Equation (1) describes the complex channel response under quasi-static structural paths and dynamic biological paths, while Equation (2) indicates that biological activity can induce temporal channel fluctuations. Therefore, RA-IWFNet does not reduce CSI to a single handcrafted variance statistic. Instead, it preserves the complex temporal channel response by constructing a CSI feature map:

(5) Xcsi,i=Pcsi(Hi)=S200×120ReHi(fm,t);ImHi(fm,t)m,t∈R200×120,

where $P_{\mathrm{csi}}\left(\cdot \right)$ denotes the complete CSI preprocessing operator and $S_{200\times 120}\left(\cdot \right)$ denotes the arrangement of the real–imaginary CSI sequence across subcarriers and time samples into a fixed-size temporal channel-variation map.

For the radar stream, Equation (3) shows that small biological displacement and radial velocity can induce phase variation and micro-Doppler-sensitive responses, while Equation (4) converts the raw radar signal into a range-dependent temporal response. Accordingly, RA-IWFNet constructs the radar feature map as

(6) $$\begin{array}{cc} X_{\mathrm{rad},i}\; & =P_{\mathrm{rad}}\left(s_{i}\right)\\ \; & =S_{100\times 40}\left({\left\{R_{i}\left(r,t\right)\right\}}_{r,t}\right)\in R^{100\times 40}, \end{array}$$

where $S_{100\times 40}\left(\cdot \right)$ denotes the arrangement of the range-dependent temporal responses into a fixed-size range–temporal feature map through temporal stacking. Equivalently, $P_{\mathrm{rad}}\left(\cdot \right)$ denotes the complete radar preprocessing operator, including one-dimensional fast Fourier transform along the fast-time dimension and temporal stacking along the slow-time dimension.

These two feature maps correspond to the physical sensing mechanisms discussed in Section 3: $X_{\mathrm{rad},i}$ preserves range–temporal micro-motion-sensitive responses while $X_{\mathrm{csi},i}$ preserves penetrative temporal channel variations. Both maps are then encoded by learnable neural encoders rather than by handcrafted physical-parameter extraction.

For the radar stream, the radar embedding is computed as $z_{i}^{\mathrm{rad}}=E_{\mathrm{rad}}\left(X_{\mathrm{rad},i}\right)$, where $E_{\mathrm{rad}}\left(\cdot \right)$ denotes the radar encoder. Similarly, the CSI embedding is computed as $z_{i}^{\mathrm{csi}}=E_{\mathrm{csi}}\left(X_{\mathrm{csi},i}\right)$, where $E_{\mathrm{csi}}\left(\cdot \right)$ denotes the CSI-specific encoder. The radar and CSI embeddings are concatenated and projected into a unified wireless embedding:

(7) $$z_{i}^{w}=\mathrm{Norm}(\phi_{w}([z_{i}^{\mathrm{rad}};z_{i}^{\mathrm{csi}}])),$$

where [·;·] denotes feature concatenation, $\phi_{w}\left(\cdot \right)$ denotes the wireless projection module, and $z_{i}^{w}\in R^{d}$ is the normalized wireless embedding.

This dual-band wireless representation combines the micro-motion sensitivity of mmWave radar and the penetrative channel-level variation captured by Wi-Fi CSI. Nevertheless, wireless-only recognition may still be affected by timber thickness, moisture variation, dielectric loss, sensor placement uncertainty, and multipath propagation. Therefore, the wireless embedding is further fused with the image embedding through the reliability-aware fusion module.

### 4.4. Reliability-Aware Image–Wireless Fusion

After the signal-model-informed feature-map construction, the image branch represents surface-level morphological evidence, and the wireless branch represents joint radar–CSI evidence derived from through-wood propagation and micro-motion-sensitive responses. The reliability-oriented gate then estimates the relative contribution of these two evidence sources for each input sample.

The core of RA-IWFNet is the reliability-oriented fusion gate. In this work, reliability is operationalized as the input-adaptive relative contribution of the image and wireless embeddings learned from the supervised classification objective. It is not a separately supervised uncertainty score or an explicit signal-quality measurement. Instead, the reliability-oriented gate estimates the relative extent to which evidence from each modality should contribute to the fused representation for a given input instance. This definition is consistent with the non-ideal inspection setting, where visual and wireless reliability may change across samples but explicit ground-truth reliability labels are not available. Therefore, the term reliability-aware in this work refers to reliability-oriented adaptive modality contribution rather than explicit probabilistic uncertainty estimation or separately supervised signal-quality prediction.

Given the normalized image embedding $z_{i}^{\mathrm{img}}$ and wireless embedding $z_{i}^{w}$, the two features are first concatenated as $u_{i}=\left[z_{i}^{\mathrm{img}};z_{i}^{w}\right]$. A learnable gate network G(·) then predicts the modality weighting logits and converts them into temperature-scaled modality weights:

(8) $$\begin{array}{cc} \ell_{i}\; & =G\left(u_{i}\right),\;\ell_{i}\in R^{2},\\ \alpha_{i}\; & =\left[\alpha_{i}^{\mathrm{img}},\alpha_{i}^{w}\right]=\mathrm{Softmax}\left(\frac{\ell_{i}}{T}\right),\\ \alpha_{i}^{\mathrm{img}}+\alpha_{i}^{w}\; & =1,\;\alpha_{i}^{\mathrm{img}}\geq 0,\;\alpha_{i}^{w}\geq 0, \end{array}$$

where the two elements of $\ell_{i}$ correspond to the image and wireless modalities, respectively; $\alpha_{i}^{\mathrm{img}}$ and $\alpha_{i}^{w}$ denote the image and wireless weights; and T>0 is the gate temperature. Therefore, the gate does not produce an unconstrained scaling factor; instead, it learns a normalized relative contribution between the image and wireless modalities for each input instance.

The pre-normalized fused representation is computed as ${\bar{z}}_{i}=\alpha_{i}^{\mathrm{img}}z_{i}^{\mathrm{img}}+\alpha_{i}^{w}z_{i}^{w}$, and the final fused embedding is obtained as $z_{i}^{\mathrm{fuse}}=\mathrm{Norm}\left({\bar{z}}_{i}\right)$. Because $z_{i}^{\mathrm{img}}$ and $z_{i}^{w}$ are projected into the same 128-D embedding space and $\ell_{2}$-normalized before fusion, the weighted summation operates on comparable feature directions. Before the final normalization, ${\bar{z}}_{i}$ can be interpreted as the minimizer of a weighted squared-distance objective:

(9) $${\bar{z}}_{i}=\arg\min_{z}\left(\alpha_{i}^{\mathrm{img}}∥z-z_{i}^{\mathrm{img}}{∥}_{2}^{2}+\alpha_{i}^{w}{∥z-z_{i}^{w}∥}_{2}^{2}\right),$$

under the unit-sum and non-negativity constraints in Equation (8). The final normalization projects the fused representation back to the normalized embedding space before classification. This formulation does not claim global optimality over all possible multimodal fusion strategies; rather, it provides a simple and interpretable feature-level mechanism for balancing two normalized modality embeddings.

This design enables the model to reduce over-reliance on a single modality. When visual observations are degraded, wireless features can provide through-wood sensing evidence. When wireless signals are disturbed, image features can provide complementary appearance cues. The reliability-oriented gate therefore allows RA-IWFNet to construct a more robust cross-modal representation when visual observations and wireless signals are jointly degraded.

### 4.5. Task-Oriented Prediction

The fused representation is passed to a classification head for four-class prediction, denoted as ${\hat{y}}_{i}=H_{\mathrm{fuse}}\left(z_{i}^{\mathrm{fuse}}\right)$, where $H_{\mathrm{fuse}}\left(\cdot \right)$ denotes the fusion classification head and ${\hat{y}}_{i}$ is the predicted class distribution. For comparison, image-only and wireless-only recognition modes are also implemented as ${\hat{y}}_{i}^{\mathrm{img}}=H_{\mathrm{img}}\left(z_{i}^{\mathrm{img}}\right)$ and ${\hat{y}}_{i}^{w}=H_{w}\left(z_{i}^{w}\right)$, respectively. These single-modality modes are used only for experimental comparison. The proposed RA-IWFNet uses the fused representation $z_{i}^{\mathrm{fuse}}$ for final prediction.

### 4.6. Training Objective

The model is trained using supervised cross-entropy loss. For a training sample with ground-truth label $y_{i}$, the overall training objective is $L_{\mathrm{total}}=L_{\mathrm{ce}}=\mathrm{CE}\left(y_{i},{\hat{y}}_{i}\right)$, where CE(·) denotes the cross-entropy loss.

The model is optimized using the supervised classification objective, while modality weighting is learned implicitly through the input-adaptive fusion gate. No separate reliability-supervision loss is introduced because explicit modality-reliability labels or signal-quality labels are not available in this inspection setting. However, the gate network is optimized end-to-end through the classification loss because the fused representation depends on the modality weights. Let $\theta_{G}$ denote the parameters of the gate network. The gradient path can be written schematically as

(10) $$\frac{\partial L_{\mathrm{ce}}}{\partial \theta_{G}}=\frac{\partial L_{\mathrm{ce}}}{\partial z_{i}^{\mathrm{fuse}}}\frac{\partial z_{i}^{\mathrm{fuse}}}{\partial {\bar{z}}_{i}}\frac{\partial {\bar{z}}_{i}}{\partial \alpha_{i}}\frac{\partial \alpha_{i}}{\partial \ell_{i}}\frac{\partial \ell_{i}}{\partial \theta_{G}}.$$

This schematic chain indicates that the modality weights can receive classification-supervision signals through the fused representation. Therefore, reliability-oriented behavior in this work refers to learned input-adaptive modality contribution, rather than explicit reliability-label supervision.

### 4.7. Inference and Gate Temperature Analysis

During inference, the input image $I_{i}$, preprocessed radar feature map $X_{\mathrm{rad},i}$, and preprocessed CSI feature map $X_{\mathrm{csi},i}$ are encoded into the image embedding $z_{i}^{\mathrm{img}}$ and wireless embedding $z_{i}^{w}$. The reliability-oriented gate then produces the fused representation $z_{i}^{\mathrm{fuse}}$ for final classification.

The default gate temperature is set to T=1.00, with T>0, and is used for the primary fusion comparison. To further examine the effect of modality weighting, we conduct a post hoc gate temperature sensitivity analysis in the experiments. Increasing *T* softens the modality weighting and encourages a more balanced image–wireless contribution. When $T\to 0^{+}$, the softmax becomes sharper and tends to select the modality with the larger logit. When T→∞, the weights approach a uniform distribution, i.e., $\alpha_{i}^{\mathrm{img}}\approx \alpha_{i}^{w}\approx 0.5$. This analysis is not a separate training objective and is not used to redefine the default model; instead, it is used to examine how the sharpness of modality weighting affects fusion robustness under joint visual and wireless degradation.

In non-ideal termite inspection scenarios, image observations may be affected by lighting disturbance, blur, compression, and occlusion, while wireless signals may be affected by attenuation, noise, and multipath propagation. Gate temperature analysis helps to reveal whether the learned gate overemphasizes one modality and whether smoother modality weighting can improve cross-modal robustness.

### 4.8. Discussion

RA-IWFNet integrates visible appearance cues and through-wood wireless sensing cues into a unified representation. The image branch provides surface-level visual evidence, while the wireless branch provides complementary penetration-based sensing information from mmWave radar and Wi-Fi CSI. The reliability-oriented gate adaptively balances these two modalities, supporting termite-related recognition under field-mixed visual degradation and wireless signal disturbance.

Compared with single-modality recognition, the proposed fusion mechanism models the relative contribution of image and wireless features. This is important for ancient timber inspection, where visual visibility and wireless propagation quality may vary substantially across inspection conditions. Therefore, RA-IWFNet provides a task-oriented multimodal sensing framework for non-destructive termite detection in heritage timber structures.

## 5. Experiments and Analyses

This section evaluates RA-IWFNet under field-motivated and synchronized image–wireless inspection settings. The experiments examine visual degradation sensitivity, wireless signal degradation sensitivity, image–wireless complementarity under joint degradation, synchronized trial-level fusion verification, and fusion mechanism behavior.

### 5.1. Experimental Protocol Overview

We built a laboratory testbed integrating a camera sensor, a 60 GHz mmWave radar, a Wi-Fi CSI sensing device, and a wooden beam containing the target region. The visual modality was acquired using a Xiaomi Smart Camera C700 with an 8-megapixel sensor (Xiaomi Corporation, Beijing, China). The mmWave modality was collected using a TI IWR6843AOPEVM antenna-on-package transceiver (Texas Instruments Inc., Dallas, TX, USA), and the CSI modality was collected using an ESP32-C5-WROOM-1 module (Espressif Systems, Shanghai, China). The radar and CSI devices were positioned 20 cm from the wooden beam to acquire through-wood wireless responses, while the camera sensor was placed 30 cm away to capture surface-level visual observations.

**Figure 3:**
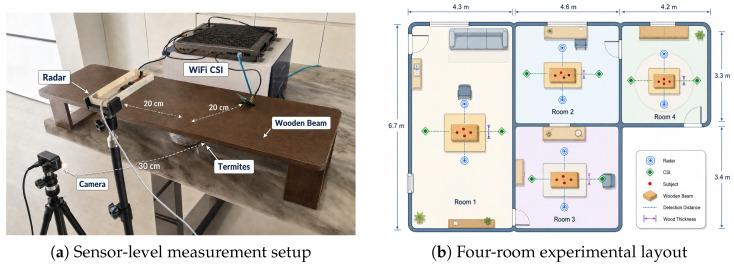
Experimental setup for image–wireless termite detection.

Figure 3 shows the sensor-level measurement setup and four-room acquisition layout. Camera images provide surface-level visual cues, whereas mmWave radar and Wi-Fi CSI provide through-wood wireless responses.

Experiments I–III were conducted under the field-motivated protocol and repeated over three independent training runs to evaluate robustness against stochastic optimization and degradation generation. Experiment IV focused on physically paired multimodal verification using the synchronized image–wireless subset and was likewise repeated over three independent training runs under the same fixed acquisition-block split. Within each run, the checkpoint with the highest validation Macro-F1 was selected for evaluation on the held-out synchronized test set.

### 5.2. Implementation Details

The image encoder was implemented using ResNet18 with a 128-D embedding. The radar and CSI streams were implemented using one-dimensional convolutional encoders followed by projection layers. Each wireless stream contains three convolutional layers with channel sizes 64, 128, and 128 and kernel sizes 5, 5, and 3, followed by batch normalization, ReLU activation, dropout, adaptive average pooling, and projection to the 128-D embedding space. The fusion gate is a lightweight multilayer perceptron that takes the concatenated image and wireless embeddings as input and outputs two modality logits. The experiments were implemented using Python 3.11 with PyTorch 2.10.0 under CUDA 12.8.

Unless otherwise specified, all models were trained using AdamW with a learning rate of $1\times 10^{-4}$. For the main field-motivated experiments, each model was reinitialized and trained from scratch over three independent runs, with the random seed controlling model initialization, data shuffling, stochastic degradation generation, and dropout. For the synchronized image–wireless verification experiment, all compared models were trained with a batch size of 32, a maximum of 40 epochs, and weight decay of $1\times 10^{-4}$. For each of the three independent runs, the checkpoint achieving the highest validation Macro-F1 was selected and evaluated once on the held-out synchronized test set under the same fixed acquisition-block split. The reported results are the mean and standard deviation across these three independent test evaluations.

### 5.3. Model Complexity

To evaluate computational feasibility, we report the parameter count, model size, and inference latency of the evaluated models. Inference time was measured on the same GPU platform using synthetic input tensors with a batch size of 32, excluding disk I/O and preprocessing. The relative cost is normalized by the image-only model.

As shown in Table 2, gated fusion introduces only a small additional modality-weighting module compared with feature concatenation, increasing the parameter count by about 0.03 M. Its inference latency remains nearly identical to feature concatenation fusion. Therefore, the performance gain of gated fusion is mainly attributed to adaptive cross-modal weighting rather than to a substantially larger backbone or higher computational cost.

The wireless-only model contains fewer parameters because radar and CSI inputs are compact temporal feature maps and can be effectively encoded using lightweight one-dimensional convolutional networks. In contrast, the image branch requires a deeper visual backbone to capture high-dimensional spatial appearance patterns from RGB observations.

### 5.4. Experimental Platform and Targets

The experimental scenario contains four recognition categories: Termite, Lyctidae, Human, and None. The three sensing modalities introduced above provide complementary surface-level and through-wood observations for these categories. The camera sensor acquires surface-level visual patterns, including pest-related appearance, wooden surfaces, human interference, and no-target background conditions. The 60 GHz mmWave radar captures fine-grained biological micro-motion responses, while the 2.4 GHz Wi-Fi CSI module provides penetrative channel-level variations through wooden obstacles.

Four classes are considered: Termite, Lyctidae, Human, and None. The Termite class denotes termite-related target conditions within the timber inspection scenario. The Lyctidae class represents a hard negative pest-related category because it is also associated with wood damage and may present visually or behaviorally similar cues to termite-related activity. The Human class represents common non-target human interference during inspection scenarios, while the None class denotes the background condition without any biological target.

After preprocessing, each radar sample is represented as $X_{\mathrm{rad}}\in R^{100\times 40}$, and each CSI sample is represented as $X_{\mathrm{csi}}\in R^{200\times 120}$. Radar responses are converted into range–temporal feature maps, while complex CSI measurements are converted into temporal channel-variation maps by separating the real and imaginary components. Each camera-sensor image is resized to 224×224 and converted into an RGB tensor before being fed into the image encoder. The image and wireless encoders project their inputs into normalized 128-D embeddings before adaptive fusion.

### 5.5. Dataset Split and Evaluation Metrics

Table 3 summarizes the dataset composition used in the field-motivated and synchronized evaluations, including the visual image pool, the field-motivated wireless split, the field-mixed visual test set, and the synchronized image–wireless subset. The field-motivated visual and wireless evaluations are class-balanced at the test stage, while the synchronized subset is used for trial-level multimodal verification.

The camera-sensor image pool was collected using the camera sensor in the laboratory experimental setup. The field-mixed visual test set includes both camera-like normal images and typical visual degradation factors, while maintaining an overall class-balanced test set. The approximate sub-condition composition is shown in Table 4.

In the main field-motivated evaluation, image and wireless inputs are paired at the class-consistent level. Specifically, samples were matched according to the same recognition class rather than the same physical acquisition trial. This protocol evaluates whether camera-sensor cues and through-wood wireless cues provide complementary evidence for the same recognition taxonomy under non-ideal conditions, but it is not treated as physically paired trial-level fusion. To evaluate fusion under physical correspondence, we further constructed a synchronized image–wireless verification subset in which each multimodal sample was collected from the same inspection trial.

**Figure 4:**
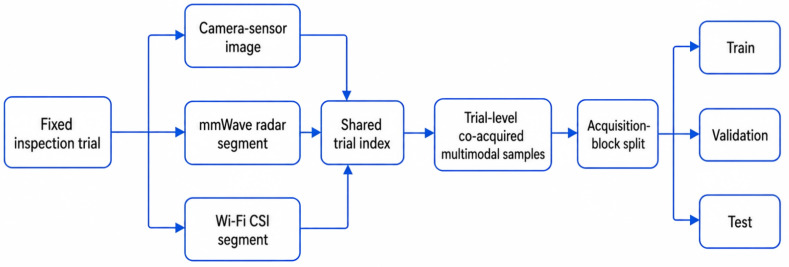
Trial-level co-acquisition and acquisition-block split protocol for the synchronized image–wireless verification subset. Records sharing the same trial index are paired as one multimodal sample before block-level dataset splitting.

Figure 4 illustrates the trial-level co-acquisition and data-splitting procedure used for the synchronized verification subset. Under each fixed inspection trial, one camera-sensor image, one mmWave radar segment, and one Wi-Fi CSI segment were collected within the same acquisition trial and assigned a shared trial index. The three modality records with the same trial index were paired as one trial-level co-acquired multimodal sample. The resulting samples were subsequently divided into training, validation, and testing subsets using an acquisition-block split.

The synchronized subset contains 2400 co-acquired multimodal samples, including 600 samples per class. For this subset, the term “synchronized” refers to controlled trial-level co-acquisition rather than frame-level synchronization. Each synchronized sample was collected under a fixed target state, timber specimen, sensor placement, and environmental setting. The image, radar, and CSI records sharing the same trial index therefore correspond to the same physical inspection trial.

During each trial, the target condition was maintained throughout the acquisition window. The camera-sensor image recorded the surface-level inspection scene, while the radar and CSI recordings captured the wireless response of the same target state. The image and wireless records were stored separately for modality-specific processing, and their correspondence was maintained through the acquisition trial index. No shared trigger or common clock was used, and correspondence across modalities was maintained through the shared acquisition trial index rather than timestamp-level alignment. This protocol represents physically paired trial-level image–wireless co-acquisition rather than frame-level synchronization, and it differs from the class-consistent pairing protocol used in Experiment III.

To reduce temporal leakage, the synchronized subset was split at the acquisition-block level, resulting in 1440 training samples, 480 validation samples, and 480 testing samples (Table 5). In this study, an acquisition block denotes a continuous recording unit collected under the same target class, timber specimen, sensor placement, environmental condition, and recording session. All samples from one acquisition block were assigned entirely to only one subset, namely training, validation, or testing. No acquisition block was shared across the training, validation, and testing subsets. This block-level split reduces the possibility that temporally adjacent wireless segments or repeated image records from the same continuous acquisition sequence appear in different subsets.

For all classification experiments, we report accuracy, Macro-F1, Termite precision, Termite recall, Termite F1-score, and pest-related confusion rates. For Experiments I–III, the Termite–Lyctidae confusion rate is computed as a bidirectional pest-related confusion rate:

(11) $$C_{T-L}=\frac{N_{T\to L}+N_{L\to T}}{\sum_{c}N_{T\to c}+\sum_{c}N_{L\to c}},$$

where $N_{T\to L}$ denotes the number of Termite samples misclassified as Lyctidae, and $N_{L\to T}$ denotes the number of Lyctidae samples misclassified as Termite.

For the synchronized verification subset, we further report the two directional confusion rates separately:

(12) $$C_{T\to L}=\frac{N_{T\to L}}{\sum_{c}N_{T\to c}},$$

(13) $$C_{L\to T}=\frac{N_{L\to T}}{\sum_{c}N_{L\to c}}.$$

For feature-space separability, the inter-class/intra-class distance ratio is computed as

(14) $$R_{\mathrm{inter}/\mathrm{intra}}=\frac{\frac{1}{K(K-1)}\sum_{a\neq b}{∥\mu_{a}-\mu_{b}∥}_{2}}{\frac{1}{K}\sum_{a=1}^{K}\frac{1}{N_{a}}\sum_{i:y_{i}=a}{∥z_{i}-\mu_{a}∥}_{2}},$$

where *K* is the number of classes, $\mu_{a}$ is the centroid of class *a*, $N_{a}$ is the number of samples in class *a*, and $z_{i}$ denotes the feature embedding of sample *i*.

### 5.6. Evaluation Protocol

Experiment I evaluates image-only recognition under field-mixed visual degradation. Experiment II evaluates wireless-only recognition under clean, mild, moderate, and severe wireless degradation. Experiment III evaluates image–wireless complementarity under joint visual and wireless degradation using the class-consistent field-motivated protocol. Experiment IV evaluates trial-level image–wireless fusion on the synchronized image–wireless verification subset. Experiment V analyzes the fusion mechanism using feature-space separability, gate-weight diagnostic statistics, gate-temperature sensitivity, and normalized confusion matrices. Primary performance comparisons in Experiments I–IV are reported over three independent training runs. Given the limited number of runs, these results are treated as descriptive repeated-run evidence rather than as formal evidence of statistical significance. Representative-checkpoint analyses are used only for diagnostic interpretation and are not treated as additional primary performance benchmarks.

### 5.7. Experiment I: Camera-Sensor Visual Degradation Sensitivity

We first evaluated image-only recognition under field-motivated visual degradation. Under the original same-domain image setting, the image-only model achieved nearly saturated performance, with 99.06% accuracy and 99.06% Macro-F1. The Termite–Lyctidae confusion rate was only 0.62%, indicating that visible pest-related images can be effectively classified under controlled visual conditions. However, this setting mainly reflects ideal visible-image recognition and does not represent field inspection of ancient timber structures, where camera-sensor acquisition may be affected by lighting disturbance, blur, compression, partial occlusion, and incomplete visual exposure. Therefore, the original same-domain result is used only as an upper-bound reference and is not treated as primary evidence of field performance.

Table 6 reports the image-only performance under the field-mixed visual condition. Results are reported as mean ± standard deviation over three independent training runs. Under field-mixed visual degradation, the image-only model achieved 68.47 ± 1.66% accuracy and 65.40 ± 2.27% Macro-F1. Although its Termite recall reached 93.45 ± 1.10%, its Termite precision was only 63.00 ± 2.26%. This indicates that field-motivated visual degradation does not merely reduce overall recognition accuracy but also changes the error pattern by increasing termite-oriented false positives. In other words, the image-only model tends to over-predict Termite when visual evidence becomes incomplete or unstable.

These results suggest that camera-sensor-only recognition is insufficient as a standalone solution under field-motivated visual degradation. Although visible images provide useful high-resolution surface-level cues, their reliability can be substantially affected by lighting variation, blurred or compressed visual details, and incomplete exposure of pest-related regions. This motivates the integration of through-wood wireless sensing, which can provide complementary activity-related information beyond surface-level visual appearance.

To further identify which field-motivated visual factors caused the degradation of image-only recognition, we divided the field-mixed test set into four camera-sensor sub-conditions: camera-like normal images, lighting disturbance, blur and compression degradation, and occlusion and cropping. This sub-condition analysis is used as a diagnostic interpretation of visual degradation patterns rather than as the primary field-performance claim. As shown in Table 7, the camera-like condition retained relatively stable performance, with 76.13% accuracy and 73.62% Macro-F1. Lighting disturbance caused a moderate decrease, reducing accuracy to 69.02%. By contrast, blur and compression degradation and occlusion and cropping led to more severe performance degradation, reducing accuracy to 57.12% and 52.61%, respectively.

The error pattern further confirms the limitation of camera-sensor-only recognition under field-motivated visual degradation. Under blur and compression degradation, Termite precision decreased to 44.00%, while Termite recall remained as high as 98.55%, indicating a strong tendency to over-predict Termite when visual details were blurred or compressed. Under occlusion and cropping, Termite precision remained low at 44.80%, and the Termite–Lyctidae confusion rate increased to 29.70%. These results show that incomplete or degraded visual cues can substantially increase false alarms and inter-class confusion, especially between visually similar pest-related categories.

### 5.8. Experiment II: Wireless-Only Signal Degradation Sensitivity

We then evaluated wireless-only recognition under different field-motivated signal quality conditions. The wireless-only model uses only mmWave radar and Wi-Fi CSI inputs, without camera-sensor visual information. Four wireless conditions were considered: clean, mild, moderate, and severe. The clean condition represents a relatively stable acquisition setting, whereas the mild, moderate, and severe conditions represent progressively lower signal quality caused by field-relevant wireless uncertainty.

In implementation, these conditions are constructed by applying progressively stronger perturbations to the preprocessed radar and CSI feature maps, including amplitude attenuation, additive noise, and partial feature loss. These perturbations approximate signal corruption that may arise from timber heterogeneity, moisture variation, dielectric loss, sensor placement uncertainty, and room-dependent multipath. The four degradation levels are not intended to isolate a single physical factor; instead, they provide a controlled protocol for evaluating robustness under increasing through-wood wireless uncertainty. The same perturbation protocol is applied consistently to all four classes to avoid class-specific bias. Table 8 summarizes the degradation parameters.

Table 9 reports the wireless-only performance under different signal degradation conditions. Results are reported as mean ± standard deviation over three independent training runs. Under the clean condition, the wireless-only model achieved 96.26 ± 1.85% accuracy and 96.26 ± 1.85% Macro-F1, indicating that mmWave radar and Wi-Fi CSI can provide effective through-wood sensing cues under relatively stable propagation conditions. However, performance decreased consistently as the wireless degradation became stronger. Under mild degradation, accuracy decreased to 87.98 ± 1.64%, while under moderate degradation it further dropped to 70.91 ± 0.33%. In the severe condition, accuracy decreased to 57.91 ± 0.13%, and Macro-F1 dropped to 57.89 ± 0.23%.

The degradation also substantially affected Termite recognition. The Termite F1-score decreased from 93.48 ± 3.21% under the clean condition to 79.83 ± 1.91%, 62.41 ± 0.96%, and 54.71 ± 0.77% under mild, moderate, and severe degradation, respectively. This trend indicates that wireless-only sensing is vulnerable to propagation instability, especially when the received signal is weakened or partially corrupted. The Termite–Lyctidae confusion rate increased from 6.49 ± 3.32% under the clean condition to 19.56 ± 2.71% under mild degradation and 28.53 ± 0.65% under moderate degradation, suggesting that degraded wireless features make it more difficult to distinguish termite activity from biologically similar interference patterns. Under severe degradation, the Termite–Lyctidae confusion rate slightly decreased to 27.30 ± 1.88%, but this does not indicate improved discriminability. Instead, the overall accuracy and Macro-F1 had already dropped substantially, suggesting that errors were more widely distributed across classes and that the discriminative quality of the wireless representation had broadly deteriorated.

These results confirm that wireless-only recognition is sensitive to complex non-ideal propagation conditions. Although mmWave radar and Wi-Fi CSI provide through-wood perception capability, their representations can still be affected by signal attenuation, noise, and multipath-related feature loss. This motivates the subsequent image–wireless complementarity experiment, where camera-sensor visual evidence is introduced to compensate for wireless uncertainty under non-ideal inspection conditions.

### 5.9. Experiment III: Image–Wireless Complementarity Under Joint Degradation

After separately analyzing the limitations of camera-sensor-only and wireless-only recognition, we further evaluated whether visual observations and wireless sensing provide complementary information under joint visual and wireless degradation. Specifically, we used the field-mixed visual condition to simulate camera-sensor inspection with lighting disturbance, blur and compression degradation, and partial occlusion or cropping. Meanwhile, the wireless signals were evaluated under the moderate degradation condition, which represents field-motivated wireless degradation through signal attenuation, additive noise, and partial feature dropout. This setting represents a practical inspection scenario in which both visual observations and wireless propagation are imperfect.

Table 10 compares the wireless-only, image-only, and image–wireless fusion models under these non-ideal conditions. Results are reported as mean ± standard deviation over three independent training runs. Under moderate wireless degradation, the wireless-only model achieved 70.91 ± 0.33% accuracy and 70.59 ± 0.37% Macro-F1, indicating that wireless sensing alone remains sensitive to propagation disturbance. Its Termite F1-score was 62.41 ± 0.96%, suggesting that degraded mmWave and Wi-Fi CSI features make it difficult to robustly identify termite-related activity.

When only field-mixed camera-sensor visual inputs were used, the image-only model achieved 68.47 ± 1.66% accuracy and 65.40 ± 2.27% Macro-F1. Although its Termite recall reached 93.45 ± 1.10%, its Termite precision was only 63.00 ± 2.26%. This result indicates that field-mixed visual observations tend to over-predict termite-related categories, leading to high recall but also a relatively large number of termite-oriented false positives.

By contrast, the fusion model achieved the highest mean performance under the same joint degradation condition, with 81.91 ± 1.33% accuracy and 81.96 ± 1.27% Macro-F1. Compared with the wireless-only model, fusion improved accuracy by 11.00 percentage points and Macro-F1 by 11.37 percentage points. The Termite F1-score also increased from 62.41 ± 0.96% to 84.00 ± 1.33%, indicating that visual cues can compensate for wireless degradation when the through-wood signal response becomes unstable. Compared with the image-only model, fusion improved accuracy by 13.44 percentage points and Macro-F1 by 16.56 percentage points. It also increased Termite precision from 63.00 ± 2.26% to 78.09 ± 0.65%, while maintaining a high Termite recall of 90.94 ± 3.70%. These results demonstrate that camera-sensor visual cues and wireless cues provide complementary evidence under the tested non-ideal inspection conditions: visual inputs provide high-resolution surface-level discrimination while wireless sensing provides through-wood activity cues that help to suppress visually induced false alarms.

To further interpret the source of improvement, we conducted a prediction-level error correction analysis using a representative trained fusion model under the class-consistent field-motivated evaluation setting. Because this diagnostic analysis is based on one representative checkpoint rather than an aggregate over multiple independent training runs, it is used only to interpret modality complementarity and is not used as the primary performance comparison. As shown in Table 11, the fusion model corrected 2340 of the 3534 samples misclassified by the image-only model, corresponding to a correction rate of 66.21%. It also corrected 1924 of the 3256 samples misclassified by the wireless-only model, corresponding to a correction rate of 59.09%. These results indicate that the fusion model does not simply follow one dominant modality but compensates for a substantial portion of modality-specific errors.

The class-specific correction results further clarify the complementary roles of the two modalities. Under field-mixed visual conditions, the image-only model produced 1538 Termite false positives, of which 896 were corrected by fusion. This suggests that wireless cues helped to suppress visual over-prediction of Termite. Conversely, under moderate wireless degradation, the wireless-only model produced 914 Termite false negatives, of which 704 were recovered by fusion. This indicates that visual information helped to restore missed Termite detections. In addition, fusion corrected 1289 of the 1600 Termite–Lyctidae confusion cases produced by the wireless-only model, showing that the fusion model substantially reduced wireless-induced confusion between the two visually and biologically similar pest categories. Together with the main performance comparison reported over three independent training runs in Table 10, these results support the complementarity of image and wireless sensing under joint visual and wireless degradation.

### 5.10. Experiment IV: Synchronized Image–Wireless Verification

Although the previous joint degradation evaluation demonstrates cross-modal complementarity under non-ideal inspection conditions, its image–wireless inputs are paired at the class-consistent level. To verify whether the proposed fusion strategy remains effective when both modalities are physically co-acquired from the same inspection trial, we further evaluated RA-IWFNet on the synchronized image–wireless verification subset described in Table 5.

It should be noted that the synchronized verification subset is not intended to replace the joint degradation evaluation in Experiment III. Experiment III evaluates robustness under simultaneous field-mixed visual degradation and moderate wireless degradation, whereas Experiment IV evaluates whether the fusion strategy remains effective when image and wireless inputs are physically co-acquired from the same inspection trial. Therefore, the results of the two experiments should not be directly interpreted as performance under the same degradation difficulty.

In this experiment, each multimodal sample contains an RGB image, an mmWave radar segment, and a Wi-Fi CSI segment acquired from the same physical inspection trial. The synchronized subset contains 2400 samples, including 600 samples for each class. To provide a controlled comparison, we evaluated five models using the same training, validation, and test split: image-only, wireless-only, feature concatenation fusion, equal-weight average fusion, and the proposed gated fusion model. All results are reported as mean ± standard deviation over three independent training runs.

Table 12 reports the performance comparison on the synchronized verification subset. The image-only model achieved 66.53 ± 0.96% accuracy and 66.42 ± 0.79% Macro-F1, indicating that visual appearance alone is insufficient for reliable termite-related discrimination in this synchronized inspection setting. The wireless-only model improved accuracy to 82.57 ± 5.95%, confirming that mmWave radar and Wi-Fi CSI provide useful subsurface sensing cues. However, wireless-only recognition still suffered from unstable Termite recognition, with a Termite F1-score of 72.81 ± 8.10%.

Compared with the single-modality baselines, the proposed gated fusion model achieved the highest mean performance, with 91.53 ± 2.44% accuracy and 91.62 ± 2.44% Macro-F1. Relative to the image-only model, gated fusion improved accuracy by 25.00 percentage points and Macro-F1 by 25.20 percentage points. Relative to the wireless-only model, it further improved accuracy by 8.96 percentage points and Macro-F1 by 9.00 percentage points. Compared with the equal-weight average fusion baseline, gated fusion also improved accuracy by 2.02 percentage points and Macro-F1 by 1.95 percentage points. These results indicate that physically co-acquired visual and wireless observations provide complementary information. The gated fusion model achieved the highest mean performance among the compared baselines, suggesting that adaptive modality weighting can be beneficial in this synchronized verification setting. However, because the number of independent runs is limited, the improvement over equal-weight average fusion should be interpreted as empirical evidence within the included baseline comparison rather than as statistically conclusive superiority.

To further clarify the contribution of the main model components, we conducted a compact component and fusion-strategy ablation on the synchronized verification subset. As shown in Table 13, the comparison separates the effects of image-only recognition, radar-only sensing, CSI-only sensing, dual-band wireless sensing, and different image–wireless fusion strategies. The radar-only and CSI-only variants evaluate whether the two wireless streams provide complementary evidence, while the concatenation, equal-weight average, and gated fusion rows evaluate whether adaptive weighting provides additional benefit beyond simple feature combination.

The ablation results show that the two wireless streams have different strengths and limitations. The radar-only branch achieved 76.60 ± 19.40% accuracy and 73.75 ± 23.48% Macro-F1, suggesting that radar micro-motion cues are informative but sensitive to training variability when used alone. The CSI-only branch produced lower but more stable overall performance, with 53.82 ± 1.07% accuracy and 47.06 ± 2.88% Macro-F1, indicating that CSI provides complementary channel-level evidence but is insufficient as a standalone representation in this setting. Combining radar and CSI in the dual-band wireless branch improved the performance to 82.57 ± 5.95% accuracy and 82.62 ± 6.16% Macro-F1, supporting the benefit of dual-band wireless sensing. Further integrating the image branch improved the results, and the proposed gated fusion model achieved highest mean performance. This indicates that the final improvement is contributed by both dual-band wireless representation learning and adaptive image–wireless fusion.

The learned modality weights provide further insight into the fusion behavior. The gated fusion model assigned a higher average weight to the wireless modality than to the image modality, with wireless and image weights of 0.623 ± 0.026 and 0.377 ± 0.026, respectively. This weighting pattern is consistent with the through-wood inspection scenario: wireless signals provide subsurface motion-sensitive information, while images provide complementary high-resolution surface-level cues. Therefore, the proposed gate does not simply average the two modalities; instead, it learns input-dependent modality contributions, with a larger average contribution assigned to the wireless modality in this synchronized subset.

It is also worth noting that simple fusion strategies did not consistently improve performance. Feature concatenation achieved 86.67 ± 3.00% accuracy, which was lower than the equal-weight average fusion baseline and substantially lower than the proposed gated fusion model. This suggests that simply increasing the feature dimension through concatenation may introduce redundant or noisy visual information. Equal-weight average fusion achieved competitive performance but remained inferior to gated fusion, indicating that fixed modality weighting is less suitable when modality contributions vary across synchronized inspection samples. Overall, the synchronized verification results provide direct evidence that the proposed image–wireless fusion strategy remains effective when multimodal inputs are physically paired at the trial level. The performance gains over the synchronized single-modality and non-adaptive fusion baselines therefore provide trial-level evidence for multimodal complementarity under physically co-acquired conditions, while Experiment III evaluates robustness under the more challenging joint visual and wireless degradation setting.

### 5.11. Experiment V: Representation and Reliability-Oriented Gate Mechanism Analysis

To further investigate the mechanisms associated with the performance improvement of the proposed fusion model, we conducted mechanism-level analyses from four perspectives: feature-space separability, gate-weight diagnostic statistics, reliability-oriented gate temperature sensitivity, and normalized confusion matrix evaluation.

We first conducted a **feature-space separability analysis** by comparing image, wireless, and fused embeddings. Here, image and wireless features refer to the modality-specific branch embeddings extracted from a representative trained fusion model before reliability-oriented feature fusion. Three metrics were used to quantify feature separability: Silhouette score, Davies–Bouldin index, and the inter-class/intra-class distance ratio.

**Figure 5:**
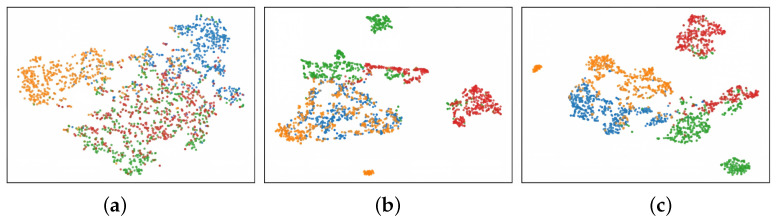
t-SNE visualization of feature spaces under field-mixed visual conditions and moderate wireless degradation. (**a**) Image feature space. (**b**) Wireless feature space. (**c**) Fused feature space. Blue, orange, green, and red points represent Termite, Lyctidae, Human, and None, respectively. The fused feature space shows clearer class-level organization than the single-modality feature spaces.

As shown in Table 14, the fused feature achieved the highest Silhouette score, the lowest Davies–Bouldin index, and the highest inter-class/intra-class distance ratio, indicating improved class compactness and separation. The t-SNE visualization in Figure 5 further supports this finding. The image feature space exhibits substantial class mixing under field-mixed visual degradation, while the wireless feature space shows partial separation but still contains overlapping regions. In contrast, the fused feature space presents clearer class-level organization, suggesting that the fusion model integrates complementary visual and wireless cues into a more separable cross-modal representation.

To further examine whether the reliability-oriented gate applies a fixed fusion ratio or produces input-dependent modality contributions, we analyzed the gate-weight distributions on the 11,200-sample fusion test set under joint visual and wireless degradation. This analysis provides a mechanism-level interpretation of adaptive modality contribution rather than an additional performance benchmark. For this gate-weight distribution analysis, gate weights were extracted from one of the independently trained fusion checkpoints used in the three-run field-motivated evaluation. The corresponding checkpoint achieved 81.04% accuracy and 81.13% Macro-F1 on the 11,200-sample fusion test set, with mean image and wireless modality weights of 0.403 and 0.597, respectively. This analysis was used only to characterize sample-wise gate behavior and does not replace the three-run aggregate performance reported in Table 10.

Table 15 reports the gate-weight statistics grouped by visual condition and target class. Because this analysis focuses on the sample-wise distribution of learned modality contributions, the reported values are calculated from a representative trained fusion model rather than aggregated across multiple independent training runs. Overall, the gate assigned a larger average contribution to the wireless modality, while the learned weights exhibited clear sample-level variation rather than remaining constant across inputs. Specifically, the image weight ranged from 0.252 to 0.644, whereas the wireless weight ranged from 0.356 to 0.748.

Across visual conditions, occlusion and cropping exhibited the lowest average image contribution and the highest average wireless contribution among the tested conditions. Although the differences across visual-condition groups were modest, this pattern suggests that the learned gate can vary its modality contributions across different visual inputs rather than maintaining a fixed fusion ratio. The gate also exhibited different modality contribution patterns across target categories. Termite and Lyctidae samples showed relatively balanced image–wireless contributions, whereas Human and None samples received larger average wireless contributions.

These results indicate that the fusion module produces input-dependent modality weights rather than a fixed image–wireless ratio. Nevertheless, this analysis should be interpreted as evidence of adaptive modality contribution rather than evidence that the learned weights represent calibrated uncertainty estimates or direct physical signal-quality measurements.

We further conducted a **reliability-oriented gate temperature sensitivity analysis** using a separate representative fusion checkpoint to examine how the sharpness of modality weighting affects fusion behavior. This post hoc analysis was conducted independently from the gate-weight distribution analysis above and is intended only to examine the sensitivity of the learned fusion mechanism to temperature scaling. The use of a separate representative checkpoint does not affect the primary three-run performance comparison. The gate temperature controls the softmax-based image–wireless weighting: a lower temperature produces sharper modality selection whereas a higher temperature produces smoother and more balanced modality weights. In this experiment, temperature scaling is used as a post hoc diagnostic analysis of the learned reliability-oriented fusion gate rather than as a separate training procedure or a new model-selection strategy.

**Figure 6:**
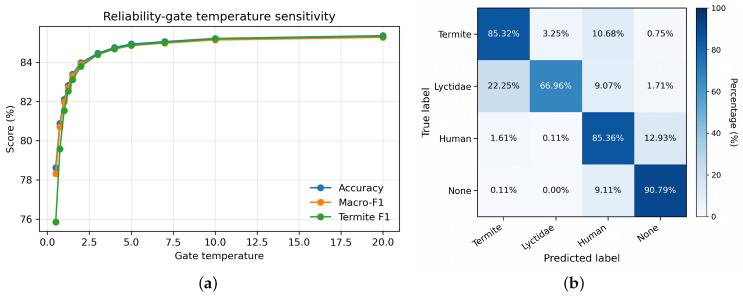
Reliability-oriented gate analysis under field-mixed visual conditions and moderate wireless degradation. (**a**) Gate-temperature sensitivity analysis. (**b**) Normalized confusion matrix of the representative default fusion model at T=1.00. Each row in the confusion matrix is normalized to percentage to show class-wise recognition accuracy.

As shown in Figure 6a, increasing the gate temperature produced higher performance on the representative fusion checkpoint within the tested range. Compared with the default setting of T=1.00, the post hoc high-temperature setting of T=20.00 increased accuracy from 82.11% to 85.36%, Macro-F1 from 81.98% to 85.27%, and Termite F1-score from 81.54% to 85.33%. Meanwhile, the mean image weight increased from 0.3321 to 0.4912, while the mean wireless weight decreased from 0.6679 to 0.5088. These observations suggest that temperature smoothing changes the balance between image and wireless contributions in the learned representation.

It should be noted that T=20.00 is not used to replace the default model configuration. This value was identified only through post hoc sensitivity analysis after the default model had been trained and selected. Therefore, the temperature analysis is used only to interpret the behavior of the learned gate and is not used to replace the three-run performance comparison reported in Table 10. Adopting a high-temperature gate as the default configuration would require a separate validation-based model-selection protocol, which will be investigated in future work.

Figure 6b reports the normalized confusion matrix of the same representative checkpoint used in the gate-temperature sensitivity analysis at the default setting of T=1.00. The default model correctly recognized 85.32% of Termite samples, 66.96% of Lyctidae samples, 85.36% of Human samples, and 90.79% of None samples. The most evident residual error occurred between the two pest-related categories, where 22.25% of Lyctidae samples were misclassified as Termite. This indicates that the main residual confusion lies between visually similar pest-related categories under timber inspection conditions, which is consistent with the difficulty of distinguishing termite-related activity from Lyctidae-related interference under field-motivated degradation.

## 6. Conclusions

This article presents RA-IWFNet, a reliability-aware image–wireless fusion network for non-destructive termite detection in heritage timber inspection. To the best of our knowledge, this work is among the first attempts to integrate camera-sensor images, mmWave radar, and Wi-Fi CSI for through-wood termite-related recognition. By combining surface-level visual cues, radar-based micro-motion responses, and CSI-based through-wood channel variations, the proposed framework formulates termite inspection as a multi-frequency wireless and image–wireless fusion sensing problem rather than as a single-modality pest-recognition task. The reliability-oriented fusion gate further enables input-adaptive balancing between image and wireless embeddings under field-motivated visual degradation and wireless signal disturbance.

Experiments on four-class recognition, including Termite, Lyctidae, Human, and None, show that RA-IWFNet achieves higher mean performance than image-only and wireless-only baselines under joint visual and wireless degradation, with 81.91 ± 1.33% accuracy and 81.96 ± 1.27% Macro-F1 over three independent training runs. On the synchronized image–wireless verification subset, gated fusion achieves 91.53 ± 2.44% accuracy and 91.62 ± 2.44% Macro-F1, yielding the highest mean performance among the compared single-modality, feature-concatenation, and equal-weight fusion baselines. Prediction-level error correction, feature-space separability, and gate-temperature analyses further indicate that the fused representation can compensate for single-modality errors and improve class separability in the tested non-ideal inspection settings.

These findings suggest that image–wireless fusion may provide a promising non-invasive pathway for termite detection in heritage timber inspection. Nevertheless, the present study should be interpreted as controlled laboratory feasibility evidence rather than full validation of field robustness in real heritage-building environments. The study remains limited by controlled degradation settings, a relatively small trial-level co-acquired verification subset, and the lack of stricter grouped-split evaluation across unseen environments. Future work will therefore extend validation to larger on-site multimodal datasets covering diverse timber properties, infestation stages, sensor geometries, and room-level clutter and multipath conditions, while further examining cross-room and cross-building generalization under stronger domain shifts.

## Figures and Tables

**Table 1:** Positioning of RA-IWFNet relative to representative multimodal fusion studies.

Method Type	Fusion Focus	Typical Modalities	Relation to This Work
Feature-level multimodal fusion [22]	Joint multimodal representation	Generic multimodal embeddings	Provides the general foundation for feature-level multimodal representation learning
Missing-modality learning [24]	Robustness to unavailable modalities	Partially missing modalities	Addresses modality absence, while this work focuses on modality degradation and complementarity
Dynamic and quality-aware fusion [25,26]	Input-dependent modality weighting	Low-quality or heterogeneous multimodal data	Motivates adaptive weighting when modality contributions vary across inputs
Adverse-condition sensor fusion [27]	Context-dependent sensor weighting	General perception sensors	Supports adaptive fusion under non-ideal sensing conditions
RA-IWFNet	Task-instantiated image–wireless adaptive fusion	Camera, mmWave radar, and Wi-Fi CSI	Integrates surface morphology and hidden wireless activity cues for termite inspection

**Table 2:** Model complexity comparison of the evaluated recognition models. Inference time is measured as average per-sample latency on the same GPU platform with a batch size of 32, excluding disk I/O and data preprocessing.

Model	Input Modality	Params (M)	Model Size (MB)	Inference Time (ms/Sample)	Relative Cost
Image-only	Image	11.26	42.99	0.379	1.00×
Wireless-only	Radar + CSI	0.32	1.22	0.040	0.11×
Feature concatenation fusion	Image + Radar + CSI	11.58	44.21	0.403	1.06×
Equal-weight average fusion	Image + Radar + CSI	11.58	44.20	0.410	1.08×
Gated fusion	Image + Radar + CSI	11.61	44.33	0.403	1.06×

**Table 3:** Dataset composition used in the field-motivated and synchronized evaluations.

Dataset/Protocol	Split or Condition	Total	Termite	Lyctidae	Human	None
Camera-sensor visual image pool	Source images	22,304	8978	3921	7958	1447
Field-motivated wireless set	Train	39,200	9800	9800	9800	9800
Field-motivated wireless set	Validation	5600	1400	1400	1400	1400
Field-motivated wireless set	Test	11,200	2800	2800	2800	2800
Field-mixed visual test set	Test	11,200	2800	2800	2800	2800
Synchronized image–wireless subset	All	2400	600	600	600	600

**Table 4:** Composition of the field-mixed visual test set.

Visual Sub-Condition	Proportion	Total	Termite	Lyctidae	Human	None
Camera-like normal images	50.75%	5684	1422	1400	1400	1462
Lighting disturbance	19.71%	2208	560	560	560	528
Blur and compression degradation	14.80%	1658	413	420	420	405
Occlusion and cropping	14.73%	1650	405	420	420	405

**Table 5:** Dataset split of the synchronized image–wireless verification subset.

Split	Total	Termite	Lyctidae	Human	None
Train	1440	360	360	360	360
Validation	480	120	120	120	120
Test	480	120	120	120	120
All	2400	600	600	600	600

**Table 6:** Image-only performance under the field-mixed camera-sensor visual condition. Results are reported as mean ± standard deviation over three independent training runs.

Visual Condition	Acc.	Macro-F1	T-Prec.	T-Rec.	T-F1
Field-mixed	68.47 ± 1.66	65.40 ± 2.27	63.00 ± 2.26	**93.45 ± 1.10**	75.24 ± 1.40

**Table 7:** Diagnostic image-only performance under different field-mixed camera-sensor sub-conditions. Results are computed using a representative trained image-only model and are used for diagnostic interpretation.

Sub-Condition	Acc.	Macro-F1	T-Prec.	T-Rec.	T-F1	T–L Conf.
Camera-like	76.13	73.62	81.17	95.78	87.87	6.27
Lighting disturbance	69.02	64.69	66.67	90.36	76.72	13.39
Blur and compression	57.12	53.00	44.00	98.55	60.84	25.21
Occlusion and cropping	52.61	46.20	44.80	90.37	59.90	29.70

**Table 8:** Wireless degradation settings used for robustness evaluation.

Condition	Amplitude Scaling	Noise std.	Feature Dropout
Clean	1.00	0.00	0.00
Mild	U(0.85,1.05)	0.03	0.03
Moderate	U(0.65,1.00)	0.08	0.08
Severe	U(0.45,0.90)	0.15	0.15

**Table 9:** Wireless-only performance under different signal degradation conditions. Results are reported as mean ± standard deviation over three independent training runs.

Wireless Condition	Acc.	Macro-F1	T-Prec.	T-Rec.	T-F1	T–L Conf.
Clean	96.26 ± 1.85	96.26 ± 1.85	92.63 ± 4.79	94.40 ± 2.41	93.48 ± 3.21	6.49 ± 3.32
Mild	87.98 ± 1.64	87.95 ± 1.62	79.57 ± 4.99	80.35 ± 3.30	79.83 ± 1.91	19.56 ± 2.71
Moderate	70.91 ± 0.33	70.59 ± 0.37	62.11 ± 1.92	62.89 ± 3.87	62.41 ± 0.96	28.53 ± 0.65
Severe	57.91 ± 0.13	57.89 ± 0.23	53.09 ± 3.46	56.73 ± 3.55	54.71 ± 0.77	27.30 ± 1.88

**Table 10:** Performance comparison under field-mixed visual conditions and moderate wireless degradation. Results are reported as mean ± standard deviation over three independent training runs.

Model	Visual	Wireless	Acc.	Macro-F1	T-Prec.	T-Rec.	T-F1
Wireless-only	–	Moderate	70.91 ± 0.33	70.59 ± 0.37	62.11 ± 1.92	62.89 ± 3.87	62.41 ± 0.96
Image-only	Field-mixed	–	68.47 ± 1.66	65.40 ± 2.27	63.00 ± 2.26	**93.45 ± 1.10**	75.24 ± 1.40
Gated fusion	Field-mixed	Moderate	**81.91 ± 1.33**	**81.96 ± 1.27**	**78.09 ± 0.65**	90.94 ± 3.70	**84.00 ± 1.33**

**Table 11:** Prediction-level error correction under field-mixed visual conditions and moderate wireless degradation. Results from a representative trained fusion model on the held-out class-consistent evaluation set are used for diagnostic analysis of modality complementarity.

Correction Case	Count	Rate
Image-only errors corrected by fusion	2340/3534	66.21%
Wireless-only errors corrected by fusion	1924/3256	59.09%
Both single-modality errors corrected by fusion	183/788	23.22%
Image Termite false positives corrected by fusion	896/1538	58.26%
Wireless Termite false negatives recovered by fusion	704/914	77.02%
Wireless T–L confusion corrected by fusion	1289/1600	80.56%

**Table 12:** Performance comparison on the synchronized image–wireless verification subset. Results are reported as mean ± standard deviation over three independent training runs. T → L denotes the Termite-to-Lyctidae confusion rate, and L → T denotes the Lyctidae-to-Termite confusion rate. (**a**) Overall and class-wise F1 performance. (**b**) Pest-related confusion and modality weights.

(a)						
**Model**	**Acc.**	**Macro-F1**	**T-F1**	**L-F1**	**H-F1**	**N-F1**
Image-only	66.53 ± 0.96	66.42 ± 0.79	71.65 ± 0.25	73.45 ± 1.43	60.30 ± 4.39	60.29 ± 1.14
Wireless-only	82.57 ± 5.95	82.62 ± 6.16	72.81 ± 8.10	85.09 ± 3.51	95.47 ± 3.28	77.11 ± 9.94
Concat fusion	86.67 ± 3.00	86.64 ± 3.11	76.72 ± 3.70	88.25 ± 3.08	99.03 ± 0.63	82.55 ± 11.23
Average fusion	89.51 ± 2.47	89.67 ± 2.37	82.50 ± 3.72	88.64 ± 0.93	**99.44 ± 0.24**	88.11 ± 5.08
**Gated fusion**	**91.53 ± 2.44**	**91.62 ± 2.44**	**84.70 ± 4.51**	**91.20 ± 1.55**	99.31 ± 0.48	**91.26 ± 3.59**
**(b)**						
**Model**	**T → L**	**L → T**	**Img W**	**Wireless W**		
Image-only	22.50 ± 4.41	12.78 ± 4.28	–	–		
Wireless-only	18.06 ± 9.59	7.22 ± 5.02	–	–		
Concat fusion	15.83 ± 9.28	7.50 ± 0.83	–	–		
Average fusion	**8.89 ± 3.94**	11.39 ± 5.91	0.500 ± 0.000	0.500 ± 0.000		
**Gated fusion**	9.44 ± 4.11	7.50 ± 1.44	0.377 ± 0.026	0.623 ± 0.026		

**Table 13:** Component and fusion-strategy ablation on the synchronized image–wireless verification subset. Results are reported as mean ± standard deviation over three independent training runs.

Variant	Image	Radar	CSI	Fusion Strategy	Acc.	Macro-F1	T-F1
Image-only branch	Yes	No	No	–	66.53 ± 0.96	66.42 ± 0.79	71.65 ± 0.25
Radar-only wireless branch	No	Yes	No	–	76.60 ± 19.40	73.75 ± 23.48	55.68 ± 33.39
CSI-only wireless branch	No	No	Yes	–	53.82 ± 1.07	47.06 ± 2.88	57.64 ± 1.84
Dual-band wireless branch	No	Yes	Yes	–	82.57 ± 5.95	82.62 ± 6.16	72.81 ± 8.10
Feature concatenation fusion	Yes	Yes	Yes	Concatenation	86.67 ± 3.00	86.64 ± 3.11	76.72 ± 3.70
Equal-weight average fusion	Yes	Yes	Yes	Fixed average	89.51 ± 2.47	89.67 ± 2.37	82.50 ± 3.72
**Gated fusion**	Yes	Yes	Yes	Adaptive gate	**91.53 ± 2.44**	**91.62 ± 2.44**	**84.70 ± 4.51**

**Table 14:** Feature-space separability comparison under field-mixed visual conditions and moderate wireless degradation.

Feature Type	Silhouette ↑	DBI ↓	Inter/Intra Ratio ↑
Image feature	0.1297	2.9331	1.3173
Wireless feature	0.1347	2.7789	1.1703
Fused feature	**0.2136**	**1.5649**	**1.4295**

**Table 15:** Gate-weight diagnostic statistics under joint visual and wireless degradation based on a representative trained fusion model. The reported values are sample-wise mean ± standard deviation within each group. The weights are interpreted as learned adaptive modality coefficients rather than calibrated reliability probabilities or explicit signal-quality estimates.

Group	Samples	Image Weight	Wireless Weight
All samples	11,200	0.403 ± 0.075	0.597 ± 0.075
Visual-condition analysis			
Camera-like normal images	5684	0.406 ± 0.081	0.594 ± 0.081
Lighting disturbance	2208	0.399 ± 0.075	0.601 ± 0.075
Blur and compression degradation	1658	0.406 ± 0.067	0.594 ± 0.067
Occlusion and cropping	1650	0.394 ± 0.064	0.606 ± 0.064
Target-category analysis			
Termite	2800	0.434 ± 0.050	0.566 ± 0.050
Lyctidae	2800	0.479 ± 0.064	0.521 ± 0.064
Human	2800	0.349 ± 0.045	0.651 ± 0.045
None	2800	0.350 ± 0.038	0.650 ± 0.038

## Data Availability

The data supporting the findings of this study are available from the corresponding author upon reasonable request.

## Abbreviations

The following abbreviations are used in this manuscript:

RA-IWFNet	Reliability-Aware Image–Wireless Fusion Network
CSI	Channel State Information
mmWave	Millimeter-wave
Wi-Fi	Wireless Fidelity
T–L	Termite–Lyctidae
Macro-F1	Macro-averaged F1-score

## References

[1] Chouvenc T. (2025). Invasive termites and their growing global impact as major urban pests. Curr. Opin. Insect Sci..

[2] Hernández-Teixidor D., Duarte S., Taheri A., Borges P.A.V., Nunes L. (2024). Molecular identification of the invasive subterranean termite *Reticulitermes grassei* (Blattodea: Rhinotermitidae) outside its known distribution: Introduction routes and implications for pest management strategies. J. Econ. Entomol..

[3] Lee C.Y., Lee S.H.D. (2025). Termite baiting—How it changed the landscape of the pest management industry and termite research in Southeast Asia. J. Econ. Entomol..

[4] Chouvenc T. (2025). How do termite baits work? Implication of subterranean termite colony demography on the successful implementation of baits. J. Econ. Entomol..

[5] Su N.Y., Mullins A., Chouvenc T. (2023). Elimination of structural and tree infestations of the Asian subterranean termite, *Coptotermes gestroi* (Wasmann) (Blattodea: Rhinotermitidae) with noviflumuron baits in above-ground stations. J. Econ. Entomol..

[6] Jiang P., Jiang L., Wu F., Che T., Wang M., Zheng C. (2025). Target Detection Method for Soil-Dwelling Termite Damage Based on MCD-YOLOv8. Sensors.

[7] Rankin G., Bui L., Tirkel A., Le Marshall N. (2012). Radar imaging: Conventional and MIMO. Proceedings of the 2012 Fourth International Conference on Communications and Electronics (ICCE).

[8] Zhang W., Zhou S., Peng D., Yang L., Li F., Yin H. (2020). Understanding and modeling of WiFi signal-based indoor privacy protection. IEEE Internet Things J..

[9] Zhang X., Zhang Y., Shi Z., Gu T. mmfer: Millimetre-wave radar based facial expression recognition for multimedia iot applications. Proceedings of the 29th Annual International Conference on Mobile Computing and Networking.

[10] Liang Y., Zhou A., Wen X., Huang W., Shi P., Pu L., Zhang H., Ma H. (2023). airBP: Monitor Your Blood Pressure with Millimeter-Wave in the Air. ACM Trans. Internet Things.

[11] Wang C., Zhao X., Li Z. (2023). Dcs-ctn: Subtle gesture recognition based on td-cnn-transformer via millimeter-wave radar. IEEE Internet Things J..

[12] Liang K., Zhou A., Zhang Z., Zhou H., Ma H., Wu C. (2023). mmStress: Distilling human stress from daily activities via contact-less millimeter-wave sensing. Proc. ACM Interact. Mob. Wearable Ubiquitous Technol..

[13] Liu X., Jiang W., Chen S., Xie X., Liu H., Cai Q., Tong X., Shi T., Qu W. (2023). PosMonitor: Fine-grained sleep posture recognition with mmWave radar. IEEE Internet Things J..

[14] Peng M., Zhao H., Fu X., Wang Y. (2024). A Multi-Orientation Real-Time Respiration Monitoring System Using WiFi Sensing Based on the Homofocal Hyperbola Model. IEEE Trans. Instrum. Meas..

[15] Han Z., Lu Z., Wen X., Zheng W., Zhao J., Guo L. (2023). CentiTrack: Toward Centimeter-Level Passive Gesture Tracking With Commodity WiFi. IEEE Internet Things J..

[16] Wang X., Niu K., Yu A., Xiong J., Yao Z., Wang J., Li W., Zhang D. (2023). WiMeasure: Millimeter-level Object Size Measurement with Commodity WiFi Devices. Proc. ACM Interact. Mob. Wearable Ubiquitous Technol..

[17] Touhiduzzaman M., Hernandez S.M., Pidcoe P.E., Bulut E. (2025). Wi-PT-Hand: Wireless Sensing based Low-cost Physical Rehabilitation Tracking for Hand Movements. ACM Trans. Comput. Healthc..

[18] Wang Z., Mao S. (2025). AIGC for wireless sensing: Diffusion-empowered human activity sensing. IEEE Trans. Cogn. Commun. Netw..

[19] Zhao C., Fang G., Ding H., Liu X., Wang F., Wang G., Zhao K., Wang Z., Xi W. (2025). Federated multi-source domain adaptation for mmwave-based human activity recognition. IEEE Trans. Mob. Comput..

[20] Teng Z., An J., Gan L., Al-Dhahir N., Han Z. (2025). Flexible intelligent metasurface for enhancing multi-target wireless sensing. IEEE Trans. Veh. Technol..

[21] McDonald J., Fitzgerald C.J., Hassan B., Morrell J. (2022). Non-destructive detection of an invasive drywood termite, Cryptotermes brevis (Blattodea: Kalotermitidae), in timber. Sociobiology.

[22] Zadeh A., Chen M., Poria S., Cambria E., Morency L.P. Tensor Fusion Network for Multimodal Sentiment Analysis. Proceedings of the 2017 Conference on Empirical Methods in Natural Language Processing.

[23] Baltrusaitis T., Ahuja C., Morency L.P. (2019). Multimodal Machine Learning: A Survey and Taxonomy. IEEE Trans. Pattern Anal. Mach. Intell..

[24] Ma M., Ren J., Zhao L., Tulyakov S., Wu C., Peng X. (2021). SMIL: Multimodal Learning with Severely Missing Modality. Proc. AAAI Conf. Artif. Intell..

[25] Zhang Q., Wu H., Zhang C., Hu Q., Fu H., Zhou J.T., Peng X. Provable Dynamic Fusion for Low-Quality Multimodal Data. Proceedings of the 40th International Conference on Machine Learning.

[26] Zadeh A.B., Liang P.P., Poria S., Cambria E., Morency L.P. (2018). Multimodal Language Analysis in the Wild: CMU-MOSEI Dataset and Interpretable Dynamic Fusion Graph. Proceedings of the 56th Annual Meeting of the Association for Computational Linguistics, Volume 1: Long Papers, Melbourne, Australia.

[27] Sural S., Sahu N., Rajkumar R. (2024). ContextualFusion: Context-Based Multi-Sensor Fusion for 3D Object Detection in Adverse Operating Conditions. Proceedings of the 2024 IEEE Intelligent Vehicles Symposium (IV).

